# Iron Catalysts in Atom Transfer Radical Polymerization

**DOI:** 10.3390/molecules25071648

**Published:** 2020-04-03

**Authors:** Sajjad Dadashi-Silab, Krzysztof Matyjaszewski

**Affiliations:** Department of Chemistry, Carnegie Mellon University, 4400 Fifth Avenue, Pittsburgh, PA 15213, USA; sdadashi@andrew.cmu.edu

**Keywords:** iron catalyst, ATRP, controlled radical polymerization, external stimuli

## Abstract

Catalysts are essential for mediating a controlled polymerization in atom transfer radical polymerization (ATRP). Copper-based catalysts are widely explored in ATRP and are highly efficient, leading to well-controlled polymerization of a variety of functional monomers. In addition to copper, iron-based complexes offer new opportunities in ATRP catalysis to develop environmentally friendly, less toxic, inexpensive, and abundant catalytic systems. Despite the high efficiency of iron catalysts in controlling polymerization of various monomers including methacrylates and styrene, ATRP of acrylate-based monomers by iron catalysts still remains a challenge. In this paper, we review the fundamentals and recent advances of iron-catalyzed ATRP focusing on development of ligands, catalyst design, and techniques used for iron catalysis in ATRP.

## 1. Introduction

Reversible deactivation radical polymerization (RDRP) techniques have provided access to advanced polymers with precise control over molecular weight, dispersity, composition, and structure. Typical approaches to control the growth of polymer chains in radical polymerizations include reversible deactivation of propagating radicals or using degenerative transfer processes to exchange radicals with dormant species in the presence of chain transfer agents. For example, atom transfer radical polymerization (ATRP) employs, primarily, Cu-based catalysts to control the growth of polymer chains via a reversible redox process that involves transfer of halogen atoms to activate dormant species generating initiating radicals and also to deactivate propagating chains [1,2,3,4]. In regard to degenerative transfer processes, reversible addition fragmentation chain transfer (RAFT) [5,6,7,8] and iodine-mediated [9,10] polymerizations have been significantly explored in controlled radical polymerizations.

ATRP catalysis has advanced based on developing new catalytic systems with the aim of progressively increasing the activity, efficiency, and selectivity of catalysts through designing ligands, using external stimuli to control the catalytic process, and also decreasing the amount of catalysts needed for achieving a controlled polymerization [11,12]. For instance, the L/Cu^I^ activator for ATRP can be generated in situ via reduction of air stable L/Cu^II^-X using external stimuli. Regeneration of the activator allows use of low concentration of the catalyst and overcoming the consumption of the activator as a result of radical termination reactions. In addition, an important aspect of using external stimuli is to exert control over the catalytic system to enable spatial and temporal control over the growth of polymer chains [13,14,15].

ATRP catalysis involves generation of radicals via activation of halogen chain ends by L/Cu^I^ activator and reversible deactivation of propagating radicals by a halogen atom transfer from L/Cu^II^-X deactivator (X = Br or Cl). While copper-based complexes have been widely explored and used for polymerization of a wide range of vinyl monomers with high efficiency, ATRP catalysis comprises other transition metal-based catalysts [16,17] such as iron [18,19,20,21], ruthenium [22], osmium [23], and iridium [24] complexes as well as organic-based photoredox catalysts [25,26,27,28].

Iron complexes are particularly interesting due to the abundance of iron and its involvement in important biological processes that make iron-based catalytic systems less toxic, inexpensive, and biocompatible. Iron complexes mainly favor one-electron redox chemistry between +2 and +3 oxidation states, suitable for ATRP catalysis. In iron-catalyzed ATRP, L/Fe^II^ species activate dormant halogen chain ends, whereas L/Fe^III^-X deactivate propagating radicals via a reversible halogen atom transfer process. Interestingly, iron complexes possessing anionic properties (for example those obtained in the presence of halide anions used as the ligand) have often performed well in ATRP reactions, whereas in copper-catalyzed ATRP, the efficient activator L/Cu^I^ complexes are cationically charged. In addition to atom transfer reactions, iron catalysts (L/Fe^II^) can also react with the propagating radicals to form organometallic species (P_n_-L/Fe^III^). While the organometallic species can be involved in an organometallic mediated radical polymerization (OMRP) mechanism [29,30], termination reactions may also be promoted through catalytic chain transfer (CCT) [31] or catalytic radical termination (CRT) processes involving P_n_-L/Fe^III^ species and propagating radicals to yield terminated chains [32]. In copper-catalyzed ATRP, CRT was reported only for the most active catalysts. Nevertheless, due to the low concentration of L/Cu^I^ present in these highly active systems, the overall contribution of OMRP and CRT processes in the polymerization is not important [33]. However, the contribution of organometallic species and CRT in iron-catalyzed systems may be more significant, leading to termination, especially in the polymerization of acrylate monomers.

In this paper, we review the principles and recent advances in iron-catalyzed ATRP. Iron catalytic systems are presented based on the structure and properties of the ligands and their effect in polymerization catalysis. Different ATRP initiating systems based on developing activator regeneration techniques and challenges and opportunities offered by iron complexes in ATRP are discussed.

## 2. Ligands and Iron Complexes in Iron-Catalyzed ATRP

Ligands play an important role in controlling the efficiency and performance of ATRP catalysts, affecting polymerization control. A variety of different families of ligands have been developed and explored in ATRP with a special focus on understanding the catalysts’ behavior in polymerization and underlying mechanisms.

### 2.1. Halide Salts

**Scheme 1:**
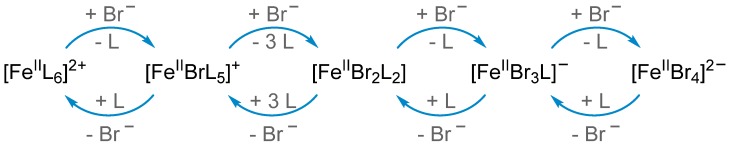
Iron species in the presence of bromide anions as ligand and solvent (L) generating a number of mononuclear cationic and anionic species in atom transfer radical polymerization (ATRP). Reproduced with slight modification and permission from Ref [43].

Halide salts with bulky, non-coordinating cations have been used as simple and robust ligands in promoting iron-catalyzed ATRP with high efficiency. Halide salts containing organic onium counter cations, ionic liquids, or inorganic based salts can be used [34,35,36,37,38,39,40,41]. Active iron complexes in the presence of halide salts possess overall anionic charge. Disproportionation (or dismutation) of Fe^II^Br_2_ forms a catalytically active anionic complex for ATRP and a cationic species [42], a much less active (or inactive) form of the catalyst, according to Equation (1):

3 Fe^II^Br_2_ → Fe^II^ + 2 [FeBr_3_]^−^ (1)

in the presence of halide salts, various iron complexes may be generated that may or may not be the active catalyst for ATRP, depending on the nature of the reaction medium and/or the amount of the ligand used. For example, iron complexes with cationic charges are less effective as ATRP catalysts, whereas anionic complexes efficiently catalyze polymerization due to the presence of high electron density around the metal center attained by complexation of anionic species (Scheme 1) [43]. Tri-coordinated monoanionic [Fe^II^Br_3_/L]^−^ (L = solvent or monomer) species was proposed to be the active species for catalyzing ATRP [39]. In the presence of excess bromide salts, dianionic [Fe^II^Br_4_]^2−^ complexes may also be generated, which contain four coordinated bromide anions rendering this species less effective to undergo a halogen abstraction during the activation of dormant chains. Therefore, the rate of polymerization decreased in the presence of excess amounts of halide salt ligands [34,35].

### 2.2. Nitrogen-Based Ligands

Alkyl amines with mono or multidentate coordinating sites or bipyridine derivatives were used for conducting iron ATRP [19,44,45,46,47]. A series of Fe^II^ complexes with *N*,*N*,*N*-trialkyl-1,4,9-triazacyclononane (TACN) ligands were investigated in ATRP [48,49,50,51]. The complexes formed either a mononuclear iron species in the presence of TACN ligands substituted with bulky groups, or trinuclear ionic species with a cationic dinuclear and an anionic mononuclear complex in the presence of less bulky substitution groups to form coordinatively saturated compounds. The mononuclear species performed well in catalyzing ATRP. Interestingly, an iron complex with cyclopentyl-substituted TACN ligand exchanged between mono and dinuclear species, therefore providing efficient catalysis and yielding well-controlled polymers, and also allowing easy purification and reusing the catalyst because of the formation of ionic structures [50].

Iminopyridine [52,53] and α-diimine [54,55,56] ligands were also developed for iron-based polymerization catalysts. Gibson and coworkers investigated the electronic and steric properties of the iron complexes with diimine ligands that affected the underlying mechanism of polymerization and therefore control over the growth of polymer chains. The electronic properties of the catalyst tuned via different *N*-substitution groups influenced the catalysts’ performance and promoted either ATRP or catalytic chain transfer (CCT) processes [31]. The CCT process was proposed to result from *N*-aryl substituted catalysts and involved formation of organometallic species followed by a hydrogen elimination to give low molecular weight, terminated polymers. *N*-Alkyl substituted complexes favored the ATRP mechanism and therefore yielded well-controlled polymers.

Bis(imino)pyridine ligands were immobilized on a polymer chain to synthesize iron catalysts for controlled polymerization [57]. The bis(imino)pyridine-containing polymer ligand was obtained using an amphiphilic copolymer comprising poly(ethylene glycol) (PEG), dodecyl, and urea/aniline pendant groups that reacted with 2,6-pyridinedicarboxaldehyde to yield self-folded polymers. Iron was immobilized on the polymer ligand and used in controlling polymerization of functional methacrylate monomers. Importantly, the amphiphilic nature of the self-folded polymers with immobilized iron catalyst allowed for easy purification of the products by rinsing with water, as the presence of hydrophilic PEG chains transferred the catalyst to aqueous phase and yielded pure polymethacrylates in the organic phase.

Shaver and co-workers developed amine-bis(phenolate) iron (III) complexes for polymerization of styrene and methacrylate monomers [58,59]. These iron complexes possess dianionic bisphenolate moieties with fixed and strong multidentate metal-ligand bonds that are less prone to undergo dissociation of the metal center during catalysis. Complexes with electron-withdrawing Cl substitution (especially in the para position of the aromatic groups) resulted in well controlled polymerizations, whereas catalysts with alkyl substituted aromatic groups gave uncontrolled results. Polymerizations were performed under reverse ATRP conditions using azobisisobutyronitrile (AIBN) as a source of radical initiator and equimolar ratios of the iron complexes. Fast and well-controlled polymerization of styrene and methacrylate monomers was obtained using the amine-bis(phenolate) iron complexes. Both Br- and Cl-based catalysts afforded well-controlled results. Control over chain growth was proposed to mainly involve the ATRP pathway as well as minor contribution of the OMRP mechanism. In the absence of ATRP alkyl halide initiators, use of an amine-bis(phenolate) iron (II) complex enabled moderate control over polymerization of methacrylate or styrene monomers via OMRP mechanism [60,61,62]. A phenolate-bis(pyridyl)amine ligand with monoanionic phenolate group was also used for iron-catalyzed ATRP but showed less efficient control over polymerization compared to the dianionic bisphenolate ligands [63].

**Scheme 2:**
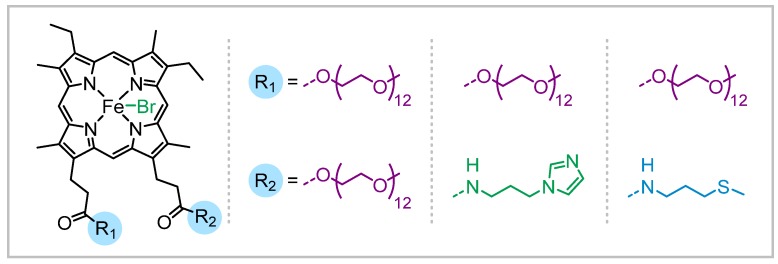
Iron porphyrin catalysts functionalized with poly(ethylene glycol) (PEG) and imidazole or thioether groups used in iron-catalyzed ATRP.

Proteins and enzymes such as horseradish peroxidase, catalase, and hemoglobin that contain iron protoporphyrin centers were used as naturally occurring, bio-inspired iron catalysts for ATRP [64,65,66,67,68]. Hemin was also used as a catalyst mimicking the enzymatic catalytic systems. Further modification of the hemin structure was performed by hydrogenation of the vinyl bonds to prevent incorporation of the catalyst in the polymer chains by addition of radicals to the double bonds. Moreover, the catalyst was functionalized by attaching PEG, imidazole, or thioether arms that enabled solubility of the catalyst in aqueous media and therefore performing well-controlled polymerizations in water (Scheme 2) [69,70,71].

### 2.3. Phosphorus-Based Ligands

**Scheme 3:**
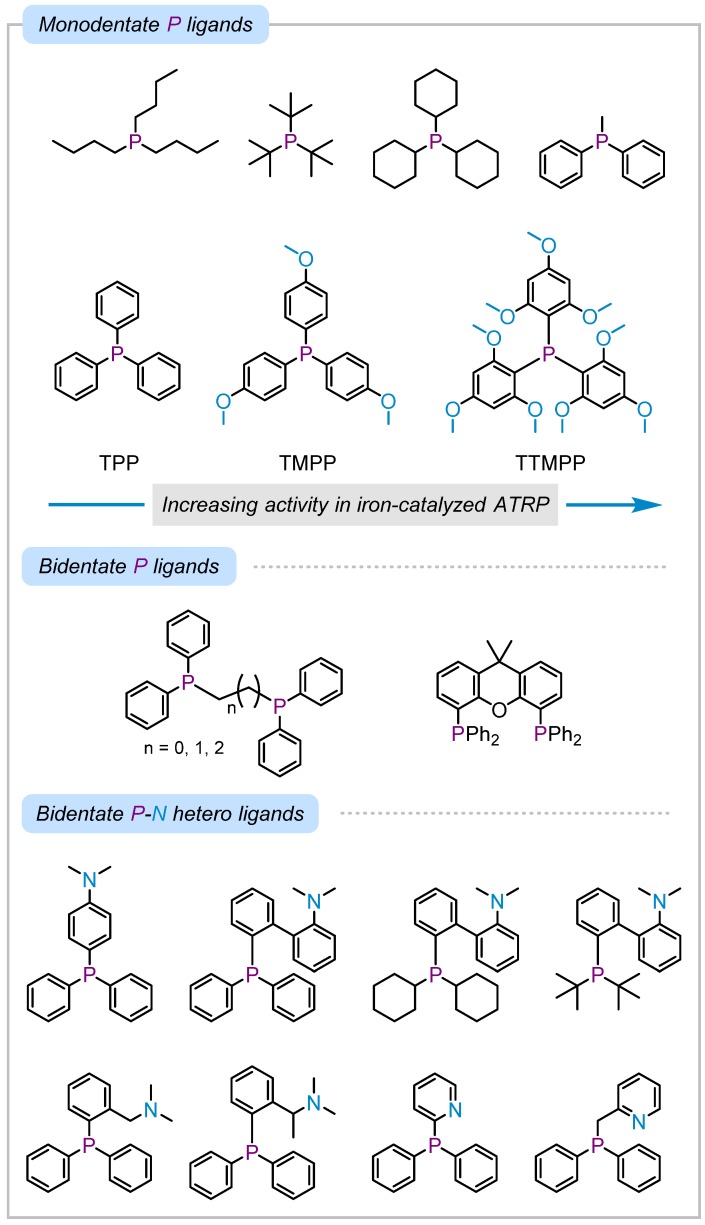
Examples of mono and bidentate phosphorus-containing ligands used in iron-catalyzed ATRP. Substitution of triphenylphosphine ligands with electron-donating methoxy groups increases the activity of the iron catalyst in ATRP.

Ligands containing phosphorus such as various alkyl and aryl compounds with mono- or bi-dentate phosphine or phosphite are among the most widely used ligands in iron-catalyzed ATRP [18,19,72,73,74,75,76]. The activity of triphenylphosphine ligands in iron-catalyzed ATRP depends on the electronic properties of the ligand, increasing in the presence of electron-donating functionalities [77]. The effect of substitution of electron donating methoxy groups on triphenylphosphine was attributed to an increase in the activity of the iron catalyst in the presence of ligands possessing more electron-donating properties, which is also reported for copper-catalyzed ATRP (Scheme 3) [78,79]. Moreover, the activity of triphenylphosphine compounds as a Lewis base increases by introducing electron donating functional groups leading to stronger reducing agents to generate L/Fe^II^ catalyst [80]. Therefore, the enhanced polymerization can be related to the electron-donating ability of the phosphine-based ligands in ATRP. In addition, similar behavior was observed in photoinduced iron-catalyzed ATRP in the presence of triphenylphosphine ligands where better control over polymerization was achieved in the presence of ligands with more electron donating properties, indicating increased activity of the iron catalyst applied in the ppm levels under blue light irradiation [81].

The effect of substitution on the phosphine-based ligands in catalyzing ATRP was further explored [77,82]. In the presence of alkyl substituted phosphines, polymerization of methyl methacrylate (MMA) was uncontrolled with high dispersity and broad or bimodal molecular weight distributions. Polymerization of MMA with triphenylphosphine was well-controlled but reached low monomer conversions. Introduction of electron donating groups in the para position of triphenylphosphine increased the activity of the resulting iron catalyst showing well-controlled polymerizations with dispersities < 1.2 and yielding high monomer conversions. The iron catalysts were obtained by reacting the phosphine ligands with FeBr_2_ for 12 h yielding diphosphine iron(II) complexes, followed by addition of monomer and initiator to start the polymerization. As expected, the iron catalyst with chloro-substituted triphenylphosphine having electron withdrawing properties was inefficient in initiating polymerization of MMA. A PEG-substituted triphenylphosphine ligand was effective in controlling polymerization of functional monomers such as hydroxyethyl methacrylate (HEMA). The iron complex with PEG-substituted triphenylphosphine tolerated functional groups due to the bulkiness of the PEG chains, which protected the catalyst center from poisoning by the functional groups. These results further signify the importance of electronic and steric properties of functional groups in designing ligands for achieving a successful and well-controlled polymerization.

In addition, bidentate ligands containing P-P homo [73,74] and P-N [83,84] or P-carbonyl [85] hetero chelating sites were reported to perform well in iron-catalyzed ATRP. The presence of a second coordinating site (P or N), increased the catalytic activity and efficiency of the iron complex. Scheme 3 shows examples of phosphorus-containing ligands developed for controlled polymerization in iron-catalyzed ATRP.

### 2.4. Miscellaneous Ligands

Iron-catalyzed ATRP can be also successfully performed in the absence of additional ligands with polar solvents that can act as a ligand for stabilizing the iron catalyst [86]. For example, iron-catalyzed ATRP was controlled in the presence of solvents such as acetonitrile, *N*,*N*-dimethylformamide (DMF), *N*-methyl-2-pyrrolidone (NMP) without the use of other ligands. These solvents resulted in disproportionation of the iron complexes to form inactive cationic species and catalytically active anionic (Equation (1) and Scheme 1) [86,87].

*N*-heterocyclic carbenes (NHC) were used as ligands in iron-catalyzed ATRP [88]. The redox potential of the iron complexes with NHC ligands were more negative than those obtained using halide salts, indicating higher activity of the catalysts with NHC ligands. As a result, control over polymerization was better with low concentrations of Fe/NHC catalysts. Interestingly, polymerizations using Br-based systems resulted in improving polymerization control compared to the Cl-based catalysts. The effect of Br vs. Cl initiating systems on control over polymerization may be related to the activity of dormant chain ends regarding the activation process, as well as halidophilicity of the iron catalyst, which influences the deactivation process and hence control over polymerization. More analysis of the Br- and Cl-based initiating systems is required to understand the effect of halide nature in the activation and deactivation processes and therefore polymerization control with iron catalysts.

## 3. Iron-Catalyzed ATRP Initiating Systems

**Scheme 4:**
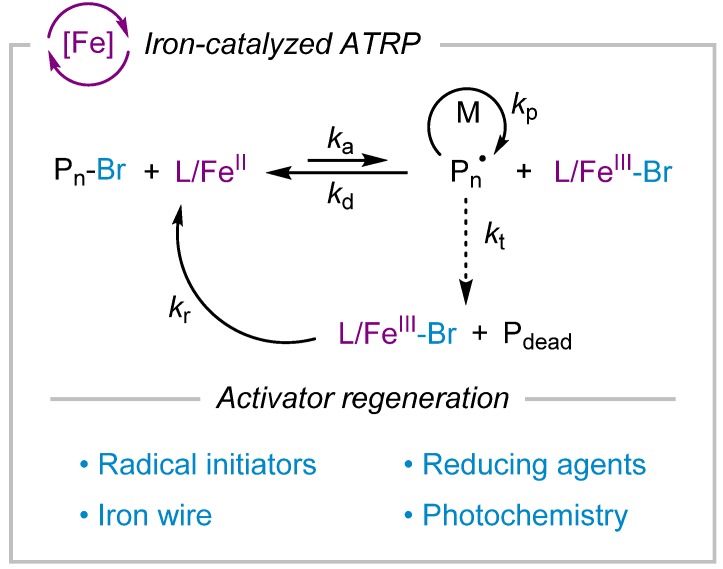
Iron-catalyzed ATRP initiating systems with activator regeneration techniques developed for generation of L/Fe^II^ activator species via reduction (*k*_r_, rate constant of reduction) of L/Fe^III^-Br.

A common feature of new ATRP initiating systems is the in situ regeneration of the activator catalyst used at ppm amount by applying various reduction processes. Regeneration of the activator species via reduction of the oxidized form of the catalyst (i.e., the deactivator) allows for use of low amounts of the catalyst starting with its more stable oxidized form, and also overcome the problems associated with the consumption of the activator as a result of termination of radicals. These techniques mainly include use of reducing agents as in activators regenerated by electron transfer (ARGET), conventional radical initiators as in initiators for continuous activator regeneration (ICAR), zerovalent metals as in supplemental activator and reducing agent (SARA) systems, or external stimuli such as photo and electrochemical approaches (Scheme 4). These systems are applicable in both copper- and iron-based ATRP reactions [38].

ICAR ATRP uses conventional thermal initiators to form radicals that can react with L/Fe^III^-X to generate the ATRP activator, L/Fe^II^ complexes, and initiate the ATRP process. Generation of radicals from decomposition of the initiator species ensures continuous activator regeneration throughout the reaction and hence results in a steady rate of polymerization and well-controlled polymers [88,89,90,91].

Reducing agents are used in ARGET ATRP to generate the activator catalyst via electron transfer processes by reducing more stable L/Fe^III^-X species [92]. Well-controlled ATRP was obtained using phosphorus ligands in the absence of conventional reducing agents. The generation of L/Fe^II^ activator was proposed to occur in the presence of phosphorus ligands that acted as a reducing agent facilitating transfer of bromine radical from L/Fe^III^-Br to the monomer and therefore generation of both the activator and the ATRP initiator in situ [77,93,94]. Decamethylferrocene (FeCp*_2_) was used as a co-catalyst to improve efficiency of polymerizations catalyzed by iron catalysts [95]. FeCp*_2_ was proposed to act as a reducing agent for FeBr_3_ in the presence of tetrabutylammonium bromide (TBABr) generating FeBr_2_ activator and the ferrocenium salt with a bromide anion. Indeed, the lower redox potential of decamethylferrocene enabled reduction of Fe^III^ to improve the kinetics of the polymerization whereas use of ferrocene having a higher redox potential than the main iron catalyst showed no effect in the polymerization. Deactivation of growing radicals was improved by contribution of the ferrocenium salt with the bromide anion.

**Scheme 5:**
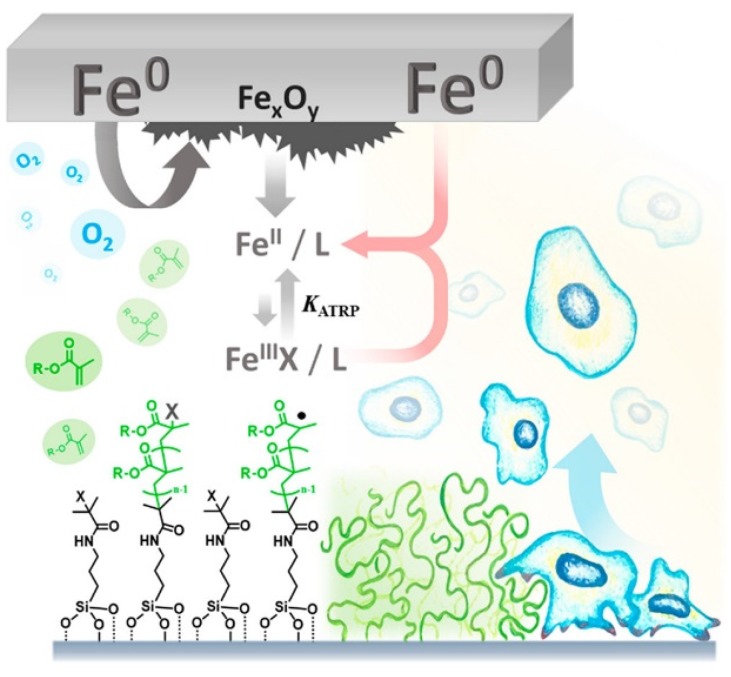
Iron-catalyzed surface-initiated ATRP using an iron plate to generate L/Fe^II^ activators and remove oxygen allowing facile surface functionalization showing high cytocompatibility. Reproduced with permission from Ref [97].

Zerovalent metal species such as iron wire or plates can be used to promote ATRP [38,96]. Recently, surface-initiated ATRP was investigated under SARA ATRP conditions using iron catalysts and an iron plate that acted as a source of activator (re)generation [97]. This surface functionalization technique was performed under ambient conditions without the need for inert atmosphere, as oxygen was consumed in the presence of iron plate, thereby simplifying the functionalization process. Importantly, the polymerization was conducted in the presence of mammalian cell cultures that showed high viability under polymerization conditions, indicating the potential of iron catalyzed ATRP to be applied under biologically relevant conditions (Scheme 5).

**Scheme 6:**
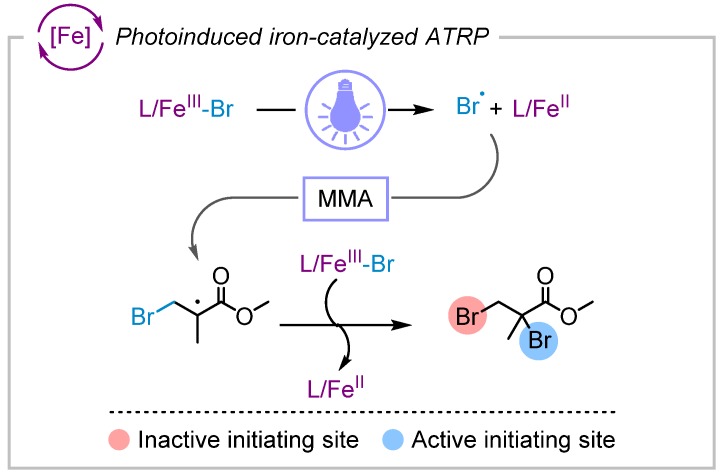
Photoinduced iron-catalyzed ATRP undergoing a homolytic cleavage of the Fe-Br bond under visible light irradiation to generate the activator L/Fe^II^ and a Br^•^ radical, which can add to the monomer (MMA) and initiate new chains [103,105]. Polymerization can be performed without the need for use of conventional alkyl halide initiators using just the monomer and the catalyst.

Photochemistry has resulted in great advancements in the field of controlled polymerizations and enabled unique opportunities for synthesis of functional polymeric materials and asserting spatiotemporal control over polymerization [98,99,100,101,102]. Photoinduced iron-catalyzed ATRP was developed for controlled polymerization of methacrylate monomers under light irradiation, which promoted generation of the L/Fe^II^ catalyst [103,104]. Photoreduction of FeBr_3_ proceeded through a homolytic cleavage of the Fe-Br bond upon photoexcitation to generate FeBr_2_ and a Br^•^ radical. The Br^•^ radical added to methacrylate monomers and generated new initiating chains. A simplified iron-catalyzed ATRP was reported by using only monomer and FeBr_3_ catalyst under UV light irradiation without using any conventional alkyl halide ATRP initiators or ligands (Scheme 6) [105]. Indeed, polymerizations relied on the formation of initiators in situ via photoreduction of FeBr_3_ generating Br^•^ radicals that reacted with methacrylate monomers. Moreover, photoinduced iron-catalyzed ATRP was further improved upon by using ppm amounts of iron catalyst while yielding well-controlled polymers under blue light irradiation [81].

Iron-catalyzed ATRP was conducted in the presence of residual oxygen to control polymerizations without performing conventional deoxygenation procedures [81]. For example, polymerization of MMA was well-controlled using 10 mol% FeBr_3_ (with respect to initiator) to give high monomer conversions (>90%) and well-controlled polymers with low dispersities (<1.20) under blue light irradiation. Importantly, block copolymers were synthesized with well-controlled properties using photoinduced iron-catalyzed ATRP in the presence of residual oxygen, indicating the potential of this system to simplify the polymerization procedure or apply in areas where no deoxygenation is desired. Although the exact mechanism of consumption of oxygen was not determined in detail, it is possible that the initiator radicals and/or the iron catalyst would contribute in removing oxygen from the solution to allow a well-controlled polymerization to proceed, as previously shown for photoinduced copper-catalyzed ATRP systems [106].

**Figure 1:**
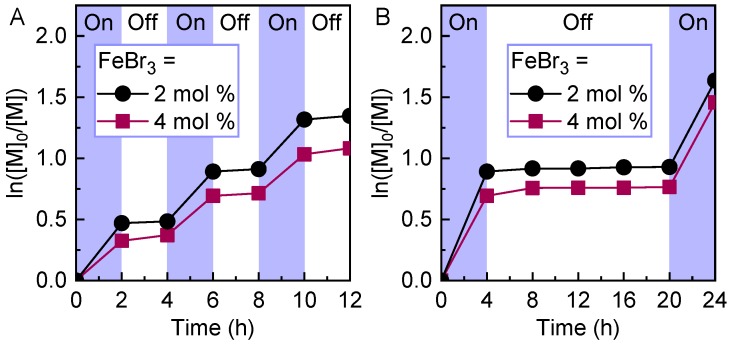
Temporal control in photoinduced iron-catalyzed ATRP of trifluoroethyl methacrylate (TFEMA). Better temporal control was achieved by decreasing the concentration of the iron catalyst from 4 to 2 mol%. Reaction conditions: [TFEMA]/[EBPA]/[FeBr_3_]/[TBABr] = 50/1/x/2x (x = 0.02 or 0.04) in 50 vol% solvent (trifluoroethanol/anisole = 9/1), irradiated under blue LEDs (465 nm, 12 mW/cm^2^). Reproduced with permission from Ref [41].

Recently, iron-catalyzed ATRP was applied for the polymerization of semi-fluorinated methacrylate monomers controlled under blue light irradiation [41]. The use of iron as a catalyst was advantageous compared to copper-based catalysts, as the use of nitrogen-ligands in these systems promoted undesired transesterification reactions between the fluorinated monomer and protic solvent leading to a loss of control over the polymerization. However, polymerization of fluorinated monomers using iron catalyst was well-controlled in the presence of a variety of fluorinated and non-fluorinated solvents without undergoing side reactions or requiring special solvent systems to control the polymerization. A variety of semi-fluorinated methacrylate monomers were polymerized in a controlled manner using FeBr_3_/TBABr as the catalyst under blue light irradiation resulting in high monomer conversions and polymers showing low dispersities (<1.20). Importantly, the use of blue light to trigger polymerization through generation of the FeBr_2_ activator catalyst enabled gaining temporal control over the growth of polymer chains. The polymerization was initiated under blue light irradiation that generated the activator catalyst. Removal of the light stopped continuous (re)generation of the activator catalyst, continuously consumed as a result of radicals’ termination, leading the polymerization to stop in the dark periods. Therefore, by decreasing the concentration of initially added catalyst, better temporal control was achieved as a result of low concentration of the activator, which required less time to be consumed during the light-off periods (Figure 1) [107].

Photoinduced iron-catalyzed ATRP was attempted in aqueous media using FeBr_3_ and water-soluble ligands (TBABr, tris[2-(2-methoxyethoxy)ethyl]amine, and triphenylphosphine-3,3′,3″-trisulfonic acid trisodium) under UV light irradiation [108]. Polymerizations in water were controlled only in the presence of high concentrations of the iron catalyst as much as 6–10 equiv. with respect to initiator (or 20000–33000 ppm with respect to monomer) giving polymers with dispersities < 1.40.

Iron complexes have been used as photocatalysts to enable ATRP under photoredox catalysis conditions [17,109]. Under visible light irradiation, the catalysts were promoted to the excited state in which the iron catalysts were effective in activating the alkyl halide initiators and generate initiating/propagating radicals. Although these iron complexes were strongly reducing in the photoexcited state (<−2.1 V), they showed very short excited state life-time of <10 ns, and therefore resulted in moderate control over polymerization.

In photoinduced ATRP, direct photolysis of the Fe-Br bond to generate the activator results in the formation of Br^•^ radicals, which are also capable of reacting with monomers and initiate polymerization of new chains. As a result, targeting high molecular weight polymers may be not accessible under these conditions, as generation of new initiating species may lead to formation of new low molecular weight chains and high dispersities. Therefore, developing photocatalytic processes where (re)generation of the activator is realized via electron or energy transfer events would be advantageous to both expand the applicability of photochemically mediated ATRP to a variety of iron complexes and also achieve well-controlled synthesis of polymers with high molecular weights.

Furthermore, electrochemically-mediated ATRP [14] has not yet been fully investigated in iron-based catalytic systems [110]. Electrochemistry would enable direct use of electrons to generate activator species without contaminating the polymer mixture with side products of the chemical reduction processes, and also better characterize the catalytic processes.

## 4. Monomer Scope in Iron-Catalyzed ATRP

Iron-catalyzed ATRP has been successfully applied in well-controlled polymerization of various methacrylate and styrene monomers. However, polymerization of monomers that contain polar functional groups can be challenging under iron-catalyzed conditions as the polar functional groups may interact with catalyst and therefore affect its catalytic efficiency.

ATRP of methacrylic acid (MAA) was controlled using heme catalysts [111]. Direct polymerization of MAA by ATRP was challenging due to termination of chain ends via lactonization. Therefore, direct ATRP of MAA required development of special conditions such as low pH to control the polymerization using a copper catalyst [112]. However, the heme catalyst was effective in controlling the polymerization of MAA to yield well-controlled polymers in aqueous solutions. Importantly, the heme-based catalysts performed well in the presence of acidic groups and in aqueous media, suggesting the potential of these compounds as robust iron based ATRP catalysts.

ATRP of other families of vinyl monomers such as acrylate and (meth)acrylamides, using iron catalysts has been challenging and less efficient. Iron-catalyzed ATRP of acrylates often leads to low monomer conversions, low initiation efficiency and polymers with high dispersities [34,55,59]. Acrylate-based secondary radicals are more susceptible to undergo formation of organometallic species with the L/Fe^II^ activators than the tertiary methacrylate-based or styrenic radicals. As a result, high degree of the formation of organometallic species and subsequent catalytic radical termination or catalytic chain transfer reactions, result in termination of polymers chains. Furthermore, the lower activity of dormant acrylate chain end compared to the methacrylate chain end may also contribute for the lower efficiency of control observed in the polymerization of acrylate monomers using iron catalysts. Considering the high propagation rate constants of acrylate monomers, the deactivation should also be fast enough to provide well-controlled polymerization. The use of iron catalysts that typically have lower activity than copper complexes may not be efficient for promoting fast initiation and activation of the chain ends in polymerization of acrylates.

ATRP of methyl acrylate (MA) using iron catalysts in the presence of halide salts gave low monomer conversions (~30%) and polymers with relatively high dispersities (1.3–1.9) [34]. Similarly, polymerization of MA using diimine iron complexes was slow and resulted in high dispersity vales (~1.5–1.6). Polymerization of butyl acrylate was well-controlled using cyclopentyl-functionalized TACN iron complexes yielding quantitative monomer conversions, and low dispersity values (1.2) [50].

An iodine-based initiating system was developed for the polymerization of acrylate monomers using iron catalysis. Dicarbonylcyclopentadienyliodoiron(II) in conjunction with a metal alkoxide such as Al(O*i*-Pr)_3_ was used in the presence of an alkyl iodide initiator to control polymerization of acrylate monomers [113]. The nature of the alkyl halide initiator greatly affected control over the polymerization as use of less active Br or Cl chain ends resulted in a loss of control with polymers having high dispersities, whereas I-based initiating system afforded well-controlled polymers with low dispersity (<1.2). The control was attributed to the synergistic effect of the iron catalyst and the metal alkoxide additive, which enhanced activation of chain ends, as well as using the more active iodine-based initiating system. However, for alkyl iodides, a degenerative transfer is also possible and can provide additional pathway for controlling polymerization [114].

Polymerization of vinyl acetate was attempted using iron(II) acetate catalyst and carbon tetrachloride (CCl_4_) as the initiator [115]. The polymerization mechanism was redox-initiated radical telomerization and not ATRP, resulting in polymers with molecular weights that did not increase with conversion but followed quite precisely [VOAc]_0_/[CCl_4_]_0_ ratios due to a C_tr_~1 for CCl_4_. Under OMRP conditions, polymerization of vinyl acetate using iron(II) acetate catalyst showed better controlled results [116].

## 5. Conclusions and Outlook

Iron complexes form an important class of ATRP catalysts that provide efficient and well-controlled polymerization of various functional monomers. There is a great potential to develop biocompatible, less toxic, and inexpensive iron-based catalytic systems for ATRP. Iron catalysts have been mainly applied for well-controlled ATRP of methacrylate and styrene monomers. Despite many successful studies on designing and use of iron catalysts in ATRP, there are still challenges that need to be addressed to take a full advantage of iron catalysis in ATRP. For instance, the use of iron catalysts has been rarely successful for controlling polymerization of acrylates, due to the possibility of formation of organometallic species and subsequent termination events. In addition to developing new iron-based catalytic systems for ATRP and broadening their utility in polymerization of new functional monomers, iron catalysts need to be explored and studied further to better understand their behavior in catalyzing ATRP reactions. For example, establishing structure-reactivity relationships in iron catalysts regarding the activation and deactivation processes would enable a better understanding of iron-catalyzed ATRP and therefore developing more efficient catalytic systems. Establishing computational approaches and high throughput experimentation would enable understanding, designing, and examining efficient iron catalysts for ATRP. Considering the biological relevance of iron compounds, it would be advantageous to develop iron-based catalytic systems, especially those derived or inspired from biological resources, for synthesis of polymer-bioconjugates or other functional polymeric materials by iron-catalyzed ATRP.
